# Pars Pro Toto: Every Single Cell Matters

**DOI:** 10.3389/fpls.2021.656825

**Published:** 2021-06-14

**Authors:** Fien Christiaens, Balkan Canher, Fien Lanssens, Anchal Bisht, Simon Stael, Lieven De Veylder, Jefri Heyman

**Affiliations:** ^1^Department of Plant Biotechnology and Bioinformatics, Ghent University, Ghent, Belgium; ^2^Center for Plant Systems Biology, VIB Center for Plant Systems Biology, Ghent, Belgium

**Keywords:** wounding, regeneration, callus, single cell, crops

## Abstract

Compared to other species, plants stand out by their unparalleled self-repair capacities. Being the loss of a single cell or an entire tissue, most plant species are able to efficiently repair the inflicted damage. Although this self-repair process is commonly referred to as “regeneration,” depending on the type of damage and organ being affected, subtle to dramatic differences in the *modus operandi* can be observed. Recent publications have focused on these different types of tissue damage and their associated response in initiating the regeneration process. Here, we review the regeneration response following loss of a single cell to a complete organ, emphasizing key molecular players and hormonal cues involved in the model species *Arabidopsis thaliana*. In addition, we highlight the agricultural applications and techniques that make use of these regenerative responses in different crop and tree species.

## Introduction: Tissue Damage From the Inside Out

Tissue damage can present itself in a wide spectrum of severity, depending on how it was inflicted. This can range from death of a single cell, a group of cells from the same or different cell type, to even loss of an entire organ. A single dead cell can arise from a spontaneously occurring event, triggered by pathogens, or can be induced artificially through laser-mediated cell ablation (Hoermayer and Friml, 2019; Marhavý et al., 2019). Similarly, death of a group of cells can be generated by both exogenous as well as endogenous factors. For example, heavy-metal-contaminated soils can trigger cell death through the induction of DNA damage (Jalmi et al., 2018), which can be mimicked by the use of radiometric DNA damage-inducing compounds, such as zeocin or bleomycin (Fulcher and Sablowski, 2009). Herbivory or harsh environmental conditions can result in tissue shearing or the loss of partial or entire organs. For each type of damage, plants have evolved elegant strategies to repair the damage sustained, for which the activated response is dictated by the type of havoc inflicted. Ultimately, complete plant bodies can be regenerated starting from tissue explants or even a single cell, including pollen, root hair cells, or protoplasts. In this review, we aim to address these different types of regeneration that have been predominantly studied in the model plant *Arabidopsis thaliana* (Arabidopsis), and highlight the possible agricultural applications in economically interesting species, including poplar, that have originated from the described responses. To conclude, we implicate the role of callus in the regeneration process, how this can be generated in an artificial setting and how this knowledge is translated in order to facilitate *in vitro* culturing and transformation of recalcitrant crop species, including maize, wheat, and rice.

## A Single Cell Is All It Takes: Death of a Single Cell

### Modes of Single Cell Replenishment

**FIGURE 1:**
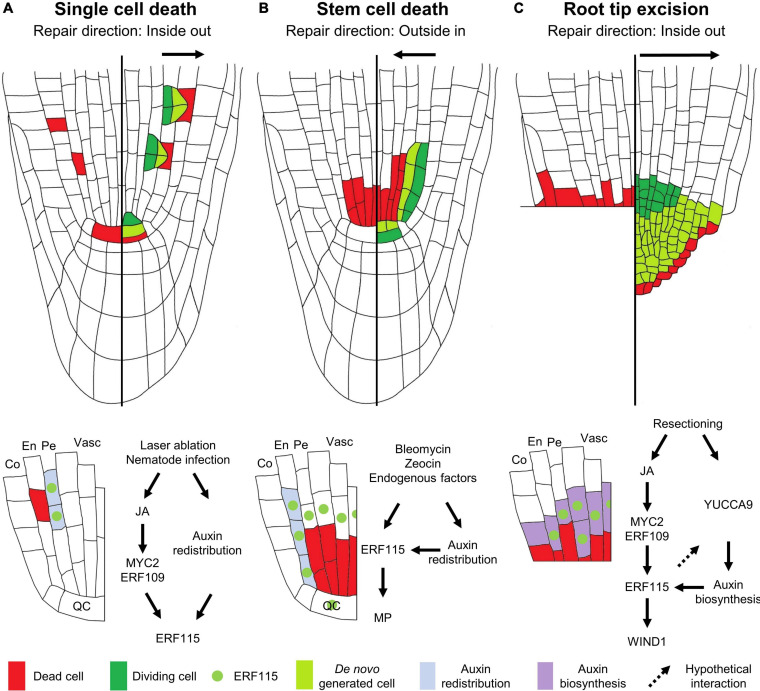
Modes of regeneration in the Arabidopsis root apical meristem following **(A)** single cell death, **(B)** vascular stem cell death, or **(C)** root tip excision. The left sides of the roots show the type of damage inflicted, the right sides illustrate the regenerative response, and the schemes below the roots indicate the molecular response. **(A)** Upon single cell damage, activated JA signaling results in the induction of the *ERF115* transcription factor (green dot) in cells neighboring the dead cell, *via* the action of MYC2/ERF109. In parallel, a local auxin response is activated in these cells (light blue). The co-occurrence of ERF115 and activated auxin signaling stimulates these cells to engage into regenerative divisions in order to replace the outward located cell. **(B)** Vascular stem cell death results in the transcriptional activation of *ERF115* in the surrounding cells, including endodermal cells and QC cells. Due to the stem cell death, the perturbed auxin flow results in the establishment of a new auxin maximum in the neighboring endodermal cells, triggering regenerative cell divisions in cells expressing *ERF115* and downstream target gene *MP*, thereby replacing the inward located cells. **(C)** Loss of the root tip, including stem cells, results in activated JA signaling that, *via* the action of MYC2/ERF109, triggers *ERF115* expression in the vascular and endodermal cells in close contact to the wound site. YUCCA9-mediated auxin biosynthesis at the wound site ensures ample auxin levels required for enforcing ERF115-mediated regenerative divisions. WIND1, directly downstream of ERF115, facilitates root tip regeneration. QC, quiescent center; En, endodermis; Co, cortex; Pe, pericycle; Vasc, vasculature.

Even loss of a single cell is sufficient to initiate a localized regenerative response. Although single cell damage can be inflicted naturally by nematodes, small insect larvae or invading necrotrophic microbes, it has been technically challenging to reproducibly target specific cells in order to study the regenerative response. However, fine-tuning of the laser-mediated single cell ablation technique allowed significant progression in our understanding of this regenerative process. In the Arabidopsis root, ablation of a single cell results in the local activation of cell division in the neighboring cells (van den Berg et al., 1995; Heyman et al., 2016; Marhavý et al., 2016; Marhava et al., 2019). Typically, cells from the contacting innermost located tissues rather than cells from the same tissue are being called upon to replenish the lost cell. This was already observed 25 years ago upon ablation of a quiescent center (QC) cell, located within the root stem cell niche (SCN) that is composed of a cluster of pluripotent cells possessing a high proliferative capacity. Laser-ablated QC cells are replaced by division of vascular stem cells that are located directly upward from the QC (van den Berg et al., 1995; Xu et al., 2006). Later, studies focusing on the tissues located higher up in the root meristem revealed a similar inside-out cell replacement mechanism. Here, loss of an endodermal cell results in the activation of cell division in the adjacent, inward located pericycle cells, which through periclinal cell division provide new cells that transdifferentiate, thereby adopting the fate of an endodermal cell (Marhavý et al., 2016; Marhava et al., 2019). In turn, loss of a cortex cell is replenished *via* periclinal cell division and subsequent transdifferentiation of the inward located neighboring endodermis cells, providing an inward-out mode of tissue regeneration (Figure 1A; Marhava et al., 2019). This mode of regeneration appears to be strongly correlated with the division potential of the responding cells. The closer the cells are to the SCN, the more efficiently a lost cell can be replaced. Venturing away from the SCN toward the end of the root meristematic zone, the regenerative potential steadily decreases, suggesting that the proliferative capacity of the cells is key for their regenerative potential (Marhava et al., 2019).

### Hormonal Response Following Single Cell Loss

Even at this single cell level, different hormones and their respective cross-talks are indispensable for damage perception and elicitation of the key downstream responses (Vega-Muñoz et al., 2020). Jasmonic acid (JA) is probably considered to be the most important wounding-responsive phytohormone, as its accumulation can be detected within seconds following damage (Glauser et al., 2009). Downstream of JA perception by the CORONATINE-INSENSITIVE PROTEIN 1 (COI1) receptor, MYC-type transcription factors, such as MYC2, initiate the expression of key downstream response genes (Figure 1A; Kazan and Manners, 2013). Following ablation of even a single root meristem cell, a swift JA response can be observed, visualized using the JAS9-VENUS reporter (Larrieu et al., 2015; Zhou et al., 2019). This JA response enables regeneration, as plants mutated for the JA receptor COI1 are unable to recover from laser-ablated QC cell loss, whereas application of JA increases the QC cell regeneration rate following ablation (Zhou et al., 2019).

Contrary, whereas the regenerative cell division response within the root meristematic zone has been proposed to be facilitated mainly by JA signaling (Zhou et al., 2019), ethylene signaling appears to be the predominant hormone involved in transmitting the single cell death signal in differentiated non-dividing root cells (Kong et al., 2018; Marhavý et al., 2019). Contrary to JA, ethylene accumulates around 30 min following wounding (Boller and Kende, 1980). Damage sustained through single cell ablation has been proposed to elicit a similar response as observed for nematode infection and can therefore be used as a proxy for studying cellular damage sustained upon nematode entry. Correspondingly, death of a single differentiated cell causes an ethylene-dominated stress response. This observation is evidenced by the robust induction of *ACS6* and *PR4*, reporting ethylene biosynthesis and signaling, respectively, following root cortical cell ablation. Contrastingly, JA does not appear to play a clear effect following mature cortex ablation, as shown by the lack of a response of the JAS9-VENUS sensor (Marhavý et al., 2019). Next to ethylene and JA, although not being a primary wound-responsive hormone, auxin plays an indispensable role during the subsequent regeneration process in the root meristematic region. Following single cell ablation, strictly localized auxin signaling, independent of biosynthesis or active transport, coordinates the regeneration response (Figure 1A). Application of the synthetic auxin 1-naphtaleneacetic acid (NAA) upon ablation increases the regenerative cell division rate and results in overproliferation of the roots, which can be interpreted as uncontrolled regenerative divisions, indicating the importance of auxin in single cell replenishment (Hoermayer et al., 2020).

### Single Cell Replenishment at the Genetic Level

To date, only a handful of genetic players have been appointed a role in the single cell regeneration process, including SCARECROW (SCR) and members of the PLETHORA (PLT) family of transcription factors. SCR represents a member of the GRAS-type family of transcription factors, involved in tissue patterning, whereas PLTs, being AP2-type transcription factors, play a predominant role in SCN specification (Shimotohno et al., 2018). It was found that plants lacking a functional SCR or a combination of PLT1 with PLT2, are unable to re-specify a new QC upon its ablation, visualized by the use of the QC-specific WUSCHEL-RELATED HOMEOBOX 5 (WOX5) marker (Haecker et al., 2004; Blilou et al., 2005), resulting in a failure to recover from the damage inflicted (Xu et al., 2006). Another key player, ETHYLENE RESPONSE FACTOR 115 (ERF115), also a member of the AP2-type transcription factors, was found to play a predominant role in the initial activation of regenerative cell divisions. Although originally identified as a rate-limiting factor controlling stress-induced QC cell divisions, *ERF115* represents an important wound-responsive gene whose activation is highly responsive to cell death (Heyman et al., 2013, 2016). It was found that death of a single root meristem cell induces *ERF115* transcription in the adjacent cells within a time frame of less than 2 h. Following its activation, ERF115 together with its interaction partner PHYTOCHOME A SIGNAL TRANSDUCTION 1 (PAT1), a member of the GRAS-type transcription factors, stimulates these cells to activate their cell division program, resulting in regenerative divisions within 5–7 h post damage (Heyman et al., 2016; Marhava et al., 2019; Zhang et al., 2019). Although auxin boosts ERF115 activity following cell ablation, application of NAA alone is not sufficient to activate *ERF115* expression (Canher et al., 2020; Hoermayer et al., 2020). In accordance with its predominant role during wound signaling, JA has been put forward to be involved in *ERF115* activation, as induction of *ERF115* could be observed in protoxylem and QC cells following JA application (Figure 1A). Furthermore, lack of *ERF115* induction upon removal of the MYC2 *cis*-regulatory element in the *ERF115* promoter revealed *ERF115* expression is activated in a MYC2-dependent way. In addition, ERF109, an ERF115 homolog and JA-responsive transcription factor, was also found to be involved in JA-dependent *ERF115* induction (Wang et al., 2008; Zhou et al., 2019). Indeed, upon laser ablation of QC cells, *ERF115* activation was reduced in seedlings defective for *COI1* (Zhou et al., 2019). Strikingly, although regenerative divisions could be detected in the cortical and epidermal cell files, *ERF115* expression was found to be confined to the endodermis and stele cells, indicating that ERF115 is not the sole factor facilitating cell replenishment and leaving the question which other factors drive the regeneration process in these tissues (Heyman et al., 2016; Marhava et al., 2019).

## A Deadpool of Cells: Repopulating a Compromized Stem Cell Niche

Environmental stresses, including hypoxia, of high light, temperature or elevated ozone levels, can trigger the generation of reactive oxygen species that in turn activate a cell death program (Van Breusegem and Dat, 2006; Beaugelin et al., 2019). Similarly, exposure of the root to high concentrations of heavy metals, such as cadmium, can result in the accumulation of dead cells in the root (Panda et al., 2008; Ye et al., 2013), probably arising due to inflicted DNA damage (Filipič, 2012; Cui et al., 2017). Likewise, it was demonstrated that when roots are exposed to near-freezing temperatures, a DNA damage-dependent apoptotic program of columella cells is executed. Sacrificing these columella cells upon exposure to low temperatures allows the release of auxin from these cells that helps to maintain quiescence and survival of the root QC (Hong et al., 2017). Additionally, endogenous factors, such as loss of MERISTEM DISORGANIZER/TEN or TOPOISOMERASE α have been shown to result in cell death, more specifically of the vascular stem cells (Hashimura and Ueguchi, 2011; Zhang et al., 2016). Such a cell death pattern can be mimicked through the application of zeocin or bleomycin (Fulcher and Sablowski, 2009) and is used to experimentally assess the activated regeneration response (Cruz-Ramírez et al., 2013; Heyman et al., 2013, 2016; Takahashi et al., 2019; Canher et al., 2020). When plants are allowed to recover from bleomycin- or zeocin-induced vascular stem cell death, regenerative divisions were originally observed in the adjacent QC cells (Cruz-Ramírez et al., 2013; Heyman et al., 2013). Whereas QC cells normally reside, as their name suggests, in a proliferation-quiescent state, they appear to be called upon in order to help to replenish the lost vascular stem cells, thereby serving as a backup pool of stem cells (Heyman et al., 2014). However, it seems unlikely that this extensive loss of vascular stem cells can be completely replenished solely by QC cell division activity. Accordingly, through time-lapse imaging and cell-lineage tracking experiments, a major contribution of the endodermal cells located directly next to the damaged vascular stem cells was observed, engaging these cells in periclinal divisions to repopulate the compromised vascular tissue (Heyman et al., 2016; Canher et al., 2020; Figure 1B). Thus, contrary to the regeneration orientation accounting for single cell replenishment, repopulation of the vascular stem cell pool occurs *via* an outside-in direction (Canher et al., 2020).

### Molecular Response

Similar to single cell damage, auxin plays an important role in orchestrating the regeneration response following loss of the vascular stem cell pool. Through a combination of cellular imaging with mathematical modeling, it was demonstrated that the activation of regenerative endodermal cell divisions correlates with a local redistribution of auxin due to a loss of auxin transporters (known as PINs), resulting in the establishment of an auxin maximum in the endodermal cells neighboring the dead vascular stem cells. The combination of a new auxin maximum with the induction of *ERF115* in the same endodermal cells, engages these cells in regenerative divisions. *MONOPTEROS* (*MP*), encoding an auxin-responsive transcription factor required for vascular development, was identified as a direct ERF115 target gene and therefore represents a key nexus point in the integration of both wound- and auxin-signaling cues for tissue regeneration (Canher et al., 2020; Figure 1B). The importance of ERF115 to allow recovery from bleomycin-induced vascular stem cell death is demonstrated by the observation that impaired ERF115 activity disenables plants to activate periclinal endodermal cell divisions, resulting in an inability to replenish the lost vascular cells. This results in a collapse of the root meristem in an upward proceeding direction (Heyman et al., 2016; Canher et al., 2020). Although JA was shown to trigger *ERF115* expression in the protoxylem tissue in the root (Zhou et al., 2019), *ERF115* induction following stem cell death could still be observed in a JA-independent way, suggesting a still yet to be identified signal that contributes to the cell death-dependent activation of *ERF115* expression (Canher et al., 2020).

## The Tip of the Regenerating Iceberg

### Regeneration of an Arabidopsis Root Tip

Regeneration of the Arabidopsis root tip following extensive tissue damage has been the topic of several studies. Over a decade ago, the first study of root tip regeneration by means of excision using a fine needle was reported by Sena et al. (2009). Upon removal of the complete root tip, including the SCN, plants were found to regenerate a *de novo* tip within three to 4 days following excision. This process requires the complete reformation of the SCN from the remaining meristematic cells. A detailed single cell transcriptomics approach combined with lineage tracking of a regenerating root tip revealed that root tip re-establishment occurs *via* rapid cell identity transitions. Here, cells from the endodermal cell files assume a stem cell-like identity in order to generate a new epidermal layer and lateral root cap. Contribution of the pericycle in the regenerating root appeared to be restricted to generating the new cortex and endodermis tissues, whereas the new SCN is derived from pre-existing stele cells adjacent to the cut site. Whereas the newly formed stem cells generate new cell files from the inside out, tissue markers revealed that cell identities are restored following an outside-in manner (Efroni et al., 2016; Figure 1C).

Identical to regenerative cell divisions following laser ablation, root tip regeneration efficiency is linked to the cell division potential of the tissues, as excision of a small root tip fragment, close to the SCN, results in a higher regeneration frequency compared to the removal of a larger tip fragment (Sena et al., 2009; Durgaprasad et al., 2019). A recent report states the presence of a regeneration competence zone, marking a clear-cut boundary beyond which no tip is able to regenerate. This competence zone appears to be marked by the presence of endogenous stem cell marker genes, such as *PLT2*, rather than the actual meristem size (Durgaprasad et al., 2019). Indeed, similarly to that observed for impaired QC cell respecification following ablation, *plt1 plt2* double mutants are largely compromized in their ability to regenerate a *de novo* root tip upon its excision (Xu et al., 2006; Sena et al., 2009).

### Molecular Components Driving Root Tip Regeneration

Similar to the previously described types of damage, excision of the root tip results in a swift *ERF115* induction that is required for regeneration, because plants with impaired ERF115 activity display a nearly complete lack of root tip regeneration potential (Heyman et al., 2016; Johnson et al., 2018; Zhou et al., 2019). Similar to that observed for single cell damage, JA appears to play a role in inducing *ERF115* upon root tip excision (Zhou et al., 2019). Directly downstream of ERF115, WOUNDING INDUCED DEDIFFERENTIATION 1 (WIND1), another member of the AP2-type of transcription factors, was identified (Heyman et al., 2016). As its name suggests, WIND1 plays a role in cellular dedifferentiation during the regeneration process (Iwase et al., 2011). Correspondingly, a role for WIND1 during root tip regeneration could be attributed (Heyman et al., 2016; Figure 1C), but surprisingly, not following single cell ablation (Marhava et al., 2019). Although being an ERF115 target gene, *WIND1* induction could be observed outside of the *ERF115* expression domain, suggesting additional mechanisms triggering WIND1-mediated cellular dedifferentiation upon wounding (Heyman et al., 2016). For example, whereas induction of both *ERF115* and *WIND1* could be observed following wounding of roots and hypocotyls, *WIND1* induction appeared to be more transient in roots, adding a putative tissue-specific preference of the regeneration response to the equation (Rymen et al., 2019).

Again, auxin is involved in root tip regeneration. However, whereas “minor” single cell damage and stem cell death result in a very localized redistribution of the available auxin pool, “extensive” loss of the entire root tip triggers a different response. Here, YUCCA9-dependent auxin biosynthesis was found to be indispensable to provide the adequate levels of auxin required to allow *de novo* root tip regeneration (Figure 1C). This auxin biosynthesis cascade appeared to be downstream of ERF115, as application of IAA was able to restore the regeneration potential of ERF115-impaired plants (Matosevich et al., 2020). The difference in auxin response upon ablation or stem cell death versus root tip excision remains an open question, but likely depends on the type of damage and corresponding intensity. Upon minor damage, the available auxin pool present in the surrounding tissues might be sufficient to initiate the regeneration program and a local redistribution will suffice. Upon stem cell death, auxin redistribution, due to the “rocks” being present in the auxin flow, triggers SCN replenishment until the normal auxin flow is re-established. However, upon more extensive damage, such as loss of the entire root tip, the main auxin biosynthesis machinery, being located in the tip, is no longer available and the remaining available auxin pool might no longer be adequate to instigate regeneration, or to respecify a novel QC, making auxin biosynthesis an essential part of the regeneration response (Canher et al., 2020).

### Regeneration of the Shoot Apical Meristem

Whereas most research has focused on the regeneration response in the Arabidopsis root tip, the response following stem cell loss in the shoot remains largely unexplored, probably due to it being experimentally less easily accessible. Although DNA damage-inducing compounds are able to induce stem cell death in the shoot (Fulcher and Sablowski, 2009), laser ablation is predominantly used to microdissect shoot stem cells. Following microdissection of the entire Arabidopsis shoot organizing center (OC), being the shoot counterpart of the root QC, a re-establishment of the OC could be detected 3 days after dissection. This is shown by the regeneration of *WUSCHEL* (*WUS*)-expressing cells, a key transcription factor marking shoot OC cells, similar to *WOX5* in the root QC cells (Mayer et al., 1998; Adibi et al., 2016). A similar laser-mediated dissection of the tomato shoot revealed that organ formation was not affected and that meristem repair was executed within 2 days, again reflected by the reformation of a *WUS*-expressing cell cluster (Reinhardt et al., 2003). Contrary to the root, re-establishment of the shoot OC is mainly driven by cytokinin signaling rather than auxin (Adibi et al., 2016).

## Cut for Repair

### Partial Incision Repair

**FIGURE 2:**
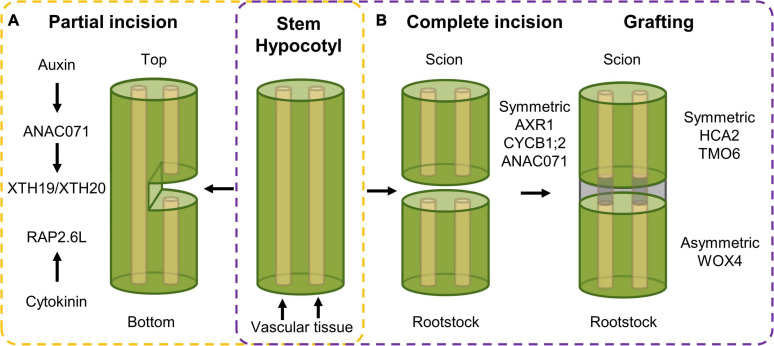
Transcriptional response following **(A)** partial incision in stem tissue and **(B)** complete incision and following tissue reuniting. **(A)** Upon partial incision of the inflorescence stem, a different response in the top versus bottom side of the cut can be observed. In the top, auxin accumulation activates expression of *ANAC071*, that in its turn induces the *XTH19* and *XTH20* cell wall modifying genes, that, in combination with cytokinin-dependent *RAP2.6L* expression in the bottom side, ensure vascular tissue reconnection. **(B)** Complete incision results in an induction of auxin-related, cell division and wound healing genes, being *AXR1*, *CYCB1;2* and *ANAC071*, respectively, in both scion and rootstock. Following grafting, symmetric and asymmetric responsive genes, being *HCA2* and *TMO6*, and *WOX4*, respectively, facilitate tissue reconnection in the hypocotyl.

Herbivore attack or harsh weather conditions can result in more extensive tissue damage, such as shearing or cuts. This type of wounding results in the activation of cell division, not in order to (re)generate tissues as such, but rather to regain a reconnection between the incised tissues. Such tissue reunion following incision requires a reactivation of cell division to allow reconnection of the severed vascular tissue, needed for water and nutrient transport throughout the plant (Figure 2A). Upon incision of the inflorescence stem, different hormonal and transcriptional changes could be observed in the top compared to the bottom part of the cut site, which together will result in tissue reunion (Asahina et al., 2011). Among these, the NAC-type ANAC071 and AP2-type RAP2.6L transcription factors are activated in order to assist in the reunion process. On the one hand, *RAP2.6L*, also known as *ERF113* and representing a homolog of *ERF115* (Heyman et al., 2018), is induced within 1 day following incision at the bottom part of the incision site in a JA-dependent manner. However, recent data using hypocotyls instead of inflorescence stems suggests that *RAP2.6L*, although being induced following a hypocotyl cut, is not required to allow cell proliferation and tissue healing to occur (Matsuoka et al., 2018). On the other hand, *ANAC071* is induced in the top part of the incision between 1 and 3 days as a result of auxin accumulation (Asahina et al., 2011). Following induction, ANAC071 activates the cell wall modifying genes *XYLOGLUCAN ENDOGLUCOSYLASE/HYDROLASE 19* (*XTH19*) and *XTH20*, which appear to catalyze the tissue reattachment process *via* hydrolysis of cell wall glucans (Pitaksaringkarn et al., 2014).

### Grafting: Tissue Reuniting Following Complete Separation

Tissue reuniting can happen as well between tissues that were completely severed, a process used in horticulture in the form of grafting, an efficient means of asexual propagation and even enabling the generation of chimeric organisms. For example, a root system (rootstock) from a plant with pathogen-resistant traits can be combined with the aerial part (scion) obtained from a plant with high crop yield, resulting in a chimeric crop having both high yield and pathogen resistance characteristics (Melnyk, 2017). It allows the combination of beneficial traits from different plant species into a single entity without the need of time-consuming breeding. Here, a similar top-versus-bottom differential response, similar to partial tissue reconnection, is observed. Transcript profiling of the bottom and top part of cut Arabidopsis hypocotyls revealed both symmetric and asymmetric gene expression responses. Genes related with auxin response [e.g., *AUXIN RESISTANT 1* (*AXR1*)], cell division [e.g., *CYCLIN B1;2* (*CYCB1;2*)], and wound healing (e.g., *ANAC071*) were activated in a symmetrical manner in grafted rootstock and scion. Contrastingly, when scion and rootstock were kept separate, the symmetrical responses were mostly preserved in the separated scion but abolished in the separated rootstock, which likely originates from the rootstock being deprived of auxin and sugars produced in the scion. Grafting-activated genes related to vascular formation can be grouped in two subsets displaying either symmetrical [e.g., *HIGH CAMBIAL ACTIVITY 2* (*HCA2*) and *TARGET OF MONOPTEROS 6* (*TMO6*)] or asymmetrical expression (e.g., *WOX4*) between the scion and the stock, referring to genes that are activated similarly in both scion and rootstock, or preferentially in only one of the two, respectively (Figure 2B). Among the symmetrically responsive genes, *HCA2* seems to be required to facilitate phloem reconnection as its impairment results in delayed reconnection rates. The asymmetrical responses peak around 72 h after grafting and disappear gradually, which is believed to result from phloem reconnection. However, genes related to the sugar response remain asymmetrically responsive even in the grafted scion and rootstocks accompanied by the formation of starch granules predominantly in the scion but also in the rootstock at later stages. Addition of exogenous sucrose lowered the grafting efficiency, pointing to the necessity of this differential sugar response between rootstock and scion, which might be important for vascular tissue reconnection (Melnyk et al., 2018). At this moment, the involvement of sugar in the tissue-reuniting process remains to be elucidated.

Recently, a major advance in grafting efficiency was found through the use of an interscion from a β-*1,4-glucanase*-overexpressing *Nicotiana benthamiana* (tobacco) plant. Here, the β-1,4-glucanase secreted from the tobacco interscion facilitates cell wall reconstruction, thereby improving cell–cell adhesion. Using this tobacco interscion as bridge, successful grafting of a tomato scion onto an Arabidopsis rootstock, otherwise graft-incompatible species, could be facilitated, again suggesting that the cell wall plays an important role in determining grafting compatibility (Notaguchi et al., 2020).

## Divide Et Impera

### Molecular Responses at the Cut Site

Whereas minor damage results in the activation of local cell divisions in order to replace the lost cells or reconnect severed tissues, such a local regeneration response will no longer suffice upon loss of an entire organ. Rather than attempting to repair or replace the lost tissue, the plant invests in generating entire *de novo* organs from the cut site, such as roots or shoots, which can be achieved directly or indirectly, but both rely on cellular reprogramming (Kareem et al., 2016). In the case of direct regeneration, cells transdifferentiate, e.g., root cells can be reprogramed to shoot cells and *vice versa*. The indirect mode relies on the generation of an intermediate regenerative mass of cells, which requires dedifferentiation of somatic cells near the wound site, allowing these cells to regain a cellular proliferation competence (Iwase et al., 2011; Kareem et al., 2016). This mass of undifferentiated, pluripotent cells is referred to as callus and serves as a base of origin from which novel organs can subsequently be formed (Stobbe et al., 2002). Although wounding-induced callus formation, generated from undifferentiated xylem cells near the wound site, is thought to prevent infection and water loss at the wound site, for example, following debarking of trees, in some cases this callus can regenerate new organs or tissues as well (Stobbe et al., 2002). For example, spontaneous wound-induced callus formation can be observed upon hypocotyl and petiole excision (Iwase et al., 2011, 2017; Liu et al., 2014).

### Rooting Following Leaf Blade Excision

**FIGURE 3:**
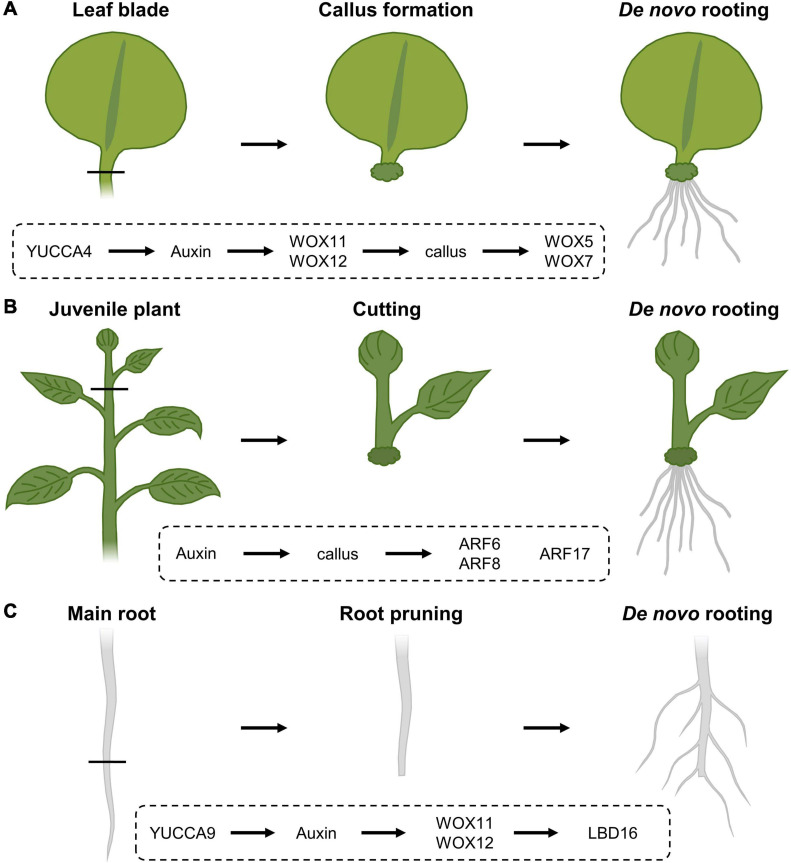
Regeneration response following **(A)** leaf blade excision, **(B)** stem cuttings and **(C)** root pruning, and the corresponding molecular cascades involved. **(A)** Upon leaf blade excision, YUCCA4-mediated auxin biosynthesis results in the induction of *WOX11* and *WOX12*, resulting in callus generation from local cambial cells. Following callus induction, WOX11 and WOX12 activate the expression of *WOX5* and *WOX7* that will initiate root primordia. **(B)** In cuttings from stems, auxin triggers callus formation and the expression of *ARF6* and *ARF8*, being positive regulators of the rooting process, as well as *ARF17*, a negative regulator of *de novo* rooting. **(C)** Following cutting of the main root system, YUCCA9-dependent auxin biosynthesis activates the expression of *WOX11* and *WOX12*, that in turn regulate *LBD16* expression, being needed to induce rooting.

Upon excision of the leaf between the blade and petiole, callus is generated locally at the cut site and adventitious roots can subsequently sprout within 8 days following excision (Figure 3A; Liu et al., 2014). Following leaf excision, auxin accumulation at the wound site, possibly provided by YUCCA4-dependent biosynthesis (Chen L. et al., 2016), directly activates expression of *WOX11*, working redundantly with *WOX12*, which enables the generation of callus from local cambium cells, that are known to contain adult stem cell populations (Lachaud et al., 1999). Root founder cells are specified from this callus within 4 days (Liu et al., 2014). Following root founder cell establishment, activity of WOX11 and WOX12 induces *WOX5* and *WOX7*, in turn initiating root primordia (Hu and Xu, 2016). However, the rooting capacity within excised leaves appears to diminish with increasing age. This “age sensing” is transmitted by SQUAMOSA PROMOTER BINDING PROTEIN-LIKE (SPL) transcription factors (Xu et al., 2016) that act as negative regulators of root regeneration by inhibiting wound-induced auxin biosynthesis (Ye et al., 2020). SPL10 appears to regulate several ERF/AP2-type transcription factor genes, including *ERF109*, being both a close homolog and putative upstream regulator of *ERF115* in the root meristem, thereby possibly imposing an age-dependent control on tissue regeneration (Heyman et al., 2018; Zhou et al., 2019; Ye et al., 2020).

### Propagation Through Cuttings

Analogously, certain tree species, including poplar, with agricultural beneficial traits can also be propagated clonally through cuttings, allowing a fast propagation of cultivars with specific traits without the need to wait for seed set or possible seed germination difficulties. Although many tree species are propagated from tissues of juvenile specimens, cloning of mature trees is generally preferred because often it is not possible to determine if selected embryos or seedlings have the genetic potential to develop the desired qualities later in their life cycle. Here, a piece of stem is excised and allowed to regenerate a *de novo* root system from the cut site (Figure 3B). Typically, the inflicted damage triggers signaling pathways that eventually result in the activation of stem cell activity at the cut site, resulting in the *de novo* formation of root primordia. Rooting on stem tissue depends on auxin, either triggered through *de novo* biosynthesis or by accumulation as a result of cutting off the basal auxin drain (Cai et al., 2014; Chen X. et al., 2016; Druege et al., 2019; Zhang et al., 2019; Matosevich et al., 2020). In Arabidopsis, early auxin maxima in etiolated hypocotyl cuttings were identified by accumulation of the *GRETCHEN-HAGEN 3-2* (*HG3-2*) auxin-response marker (Sukumar et al., 2013). Subsequent rooting is promoted by the application of indole-3-butyric acid (IBA), being an endogenous auxin, and is always preceded by callus formation (Ludwig-Müller et al., 2005). Here, the auxin maxima result in the activation of the AUXIN RESPONSE FACTOR 6 (ARF6) and ARF8, being positive regulators of the rooting process, together with ARF17, a negative regulator (Gutierrez et al., 2012). The combination of hormonal input and a complex molecular network of genetic and epigenetic changes (Jing et al., 2020) likely explains why rooting efficiency not only depends on the species and genotype, but also on growth conditions and seasonality, as well as the decrease in *de novo* rooting potential over age (Batista et al., 2015). Because of the observed drop in regeneration potential over age, even the propagation of good rooting species can become problematic over time. Such loss of competence for *de novo* rooting represents a frequently occurring issue in breeding programs, impairing the propagation of genotypes with interesting attributes for commercial production. Although several hormone-containing rooting compounds are commercially available in order to boost a cutting’s rooting potential, several agriculturally important crops or ecotypes remain recalcitrant to *de novo* root initiation.

### Root Pruning: Cut a Root to Make a Root

The regenerative response where organs are formed *de novo* following injury or even arising from the cut site, is similarly widely used in agriculture. One of these applications relies on the mechanical removal of a large mass of roots in order to stimulate formation of a denser lateral root network (Figure 3C). This process is commonly referred to as root pruning and has been shown in Arabidopsis to be facilitated, at least in part, by the action of WOX11 and again its partially redundant WOX12. Although lateral root initiation in seedlings grown under standard tissue-culturing conditions occurs independently of WOX11/12, the primary root is able to produce both WOX11-dependent and -independent roots when grown in soil or following wounding through direct transcriptional activation of *LATERAL ORGAN BOUNDARIES DOMAIN 16* (*LBD16*). Although the WOX11-mediated lateral root initiation appears to be independent of the developmentally controlled lateral root production by AUXIN RESPONSE FACTOR 7 (ARF7) and ARF19, both pathways appear to converge on LBD16 in order to initiate rooting (Lee et al., 2009; Goh et al., 2012; Sheng et al., 2017). Following removal of the main root, generation of a new root system appears to depend on the elevation of endogenous auxin levels, as the formation of this root system is abolished in mutants defective in YUCCA9-mediated auxin biosynthesis or in the presence of the polar auxin transport inhibitor naphthylphthalamic acid, commonly known as NPA (Xu et al., 2017).

In agriculture, root pruning is usually performed for economically important species such as tomato, potato, soybean, and apple trees and often stimulates a variety of phenotypic responses, depending on the species. For example, for fruit trees, in addition to increased lateral root formation, root pruning results in an increased number of flower buds, the production of smaller fruit with a higher quality, a reduction in pre-harvest fruit drop and an overall increased plant size and vigor. In potato, root pruning 2 weeks prior to harvesting leads to the formation of a firmer skin around the potatoes, resulting in a longer shelf life. In tree species like poplar, root pruning is mostly performed to create a denser mass of lateral roots, leading to a reduced shock when the plant is transported to another location, but also to expand the absorption area of the root and to improve the rhizosphere soil fertility (Jing et al., 2017, 2018).

## Coming Full Circle, Once More a Single Cell Is All It Takes

### Molecular Insights Into Callus Formation and Shoot Regeneration

**FIGURE 4:**
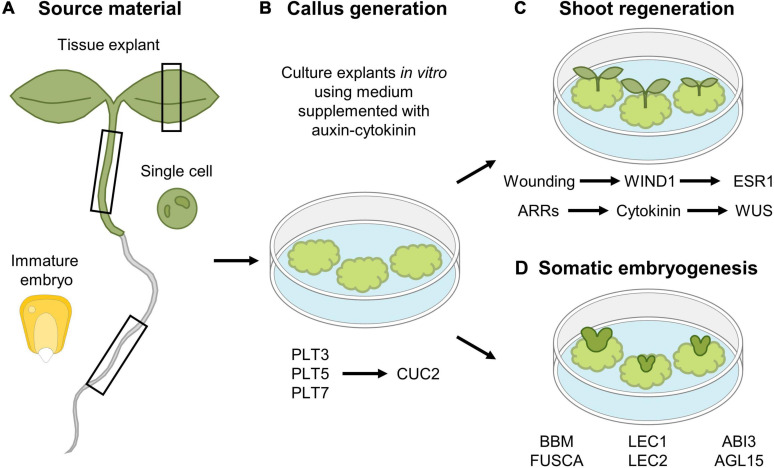
*In vitro* tissue culturing using **(A)** various tissue explants to **(B)** generate callus and subsequent **(C)** shoot regeneration or **(D)** somatic embryogenesis. **(A,B)** Different tissue explant material can be used for *in vitro* callus generation, including leaf, hypocotyl and root explants, immature maize embryos and single cultured cells. **(C)** Shoot regeneration occurs in a two-step mechanism, where callus is first generated by the action of the PLT3, PLT5, and PLT7 transcription factors, whereas in a second step, expression of *CUC2* is required for shoot induction. In parallel, a new shoot organizing center is established by the action of ARRs that facilitate cytokinin production, that in turn activates *WUS* expression. In addition, wounding-induced expression of *WIND1* allows in turn induction of *ESR1* that contributes to regeneration of shoots. **(D)** Somatic embryogenesis from callus occurs by the expression of a network of transcription factors, composed of *BBM*, *LEC2*, *FUSCA*, *ABI3*, and *AGL15*.

Besides spontaneously being generated following wounding, callus can also be generated artificially through the exogenous application of hormones, which is often the preferred *modus operandi* to study the callus formation process and which is extensively used in laboratory conditions and biotechnical applications, including plant transformation. Using this *in vitro* approach, callus can be generated from different tissue types, including leaves, cotyledons, hypocotyls, root explants, or even single cultured cells (Figure 4A). However, callus induced through wounding does not appear to display the same molecular and physiological properties compared to tissue-culture generated callus. Regardless of the source explant tissue used, hormone-induced callus rather contains root-like properties, as suggested by the expression of root meristem marker genes and the inability to be generated from mutants defective in lateral root initiation, which is not observed for wound-induced callus (Sugimoto et al., 2010; Iwase et al., 2011; Ikeuchi et al., 2013). By placing explants, or even single differentiated cells (Steward et al., 1958; Nagata and Takebe, 1971), on culture medium supplemented with a specific auxin-cytokinin phytohormone ratio, a pool of pluripotent cells is generated (Skoog and Miller, 1957), a process that is preceded by extensive epigenetic reprograming (Iwase et al., 2017; Kim et al., 2018; Rymen et al., 2019). Once the callus-like tissue is formed, roots or shoots can subsequently be induced by transferring the callus to medium supplemented with a high or low auxin-to-cytokinin ratio, respectively. In Arabidopsis, *de novo* shoot induction is facilitated in a two-step mechanism. In a first step, the PLT transcription factors PLT3, PLT5, and PLT7 are required for the induction of the root stem cell regulators PLT1 and PLT2. In a next step, PLT3/5/7 regulate the required shoot-promoting-factor CUP-SHAPED COTELYDON2, being a NAC-type transcription factor, again highlighting the importance of PLTs in callus formation and subsequent organ induction (Kareem et al., 2015; Figure 4B). Besides the role for PLTs, the induction of *WUS* is indispensable to allow shoot regeneration from callus. By placing callus on shoot-inducing medium, activity of ARABIDOPSIS RESPONSEREGULATORS (ARRs), such as ARR12, facilitate the transduction of the cytokinin signal, which is indispensable for *WUS* induction and subsequent shoot regeneration (Boutilier et al., 2002; Edgar et al., 2002; Dai et al., 2017; Meng et al., 2017; Figure 4C).

ENGHANCER OF SHOOT REGENERATION 1 (ESR1) and its paralog ESR2 were identified as key regulators of shoot formation, as ectopic expression of either *ESR1* or *ESR2* is sufficient to trigger shoot regeneration (Banno et al., 2001; Iwase et al., 2017). Recently, it was found that WIND1 acts as a direct transcriptional activator of *ESR1*, indicating that the wound-induced expression of *WIND1*, and subsequent *ERS1* activation, is required to initiate shoot regeneration (Iwase et al., 2017; Figure 4C).

The knowledge concerning this *de novo* shoot formation following callus induction was translated into an elegant tool allowing the generation of CRISPR-edited plants from tobacco leaves (Maher et al., 2020). Here, wound-free callus is generated from tobacco leaves by Agrobacterium-mediated introgression of callus-inducing factors, together with a CRISPR-Cas9 editing module. The subsequent regeneration of shoots from this edited callus allows a rapid generation of genome-edited tobacco plantlets.

### Somatic Embryogenesis and Epigenetic Control of Regeneration

Although callus formation represents a naturally occurring response to wounding or can be induced artificially through hormone supplementation, callus can also be obtained through ectopic expression of key regeneration-driving or stem cell-specific transcription factor-encoding genes, including the aforementioned *WIND1* (Iwase et al., 2011), *ERF115* in combination with its dimerization partner *PAT1* (Heyman et al., 2016), or *PLTs* such as *BABYBOOM* (*BBM*) (Boutilier et al., 2002). Contrary to the use of WIND1 and ERF115-PAT1 in the generation of callus, likely resulting from uncontrolled cellular dedifferentiation or regenerative divisions, respectively, BBM can be used to boost the *in vitro* tissue-culturing potential. Here, rather than the generation of a mass of undifferentiated cells, BBM contributes to the generation of complete plant bodies originating from only a small group of cells or even a single cell, which very well may represent the “ultimate” regeneration response, commonly known as somatic embryogenesis (Figure 4D; Fehér, 2019; Ikeuchi et al., 2019). In Arabidopsis, a transcription factor network composed of BBM, LEAFY COTYLEDON 1 (LEC1), LEC2, FUSCA, ABSCISIC ACID INSENSITIVE3, and AGAMOUS-LIKE15 has been shown to play a central role during somatic embryogenesis (Radoeva and Weijers, 2014; Horstman et al., 2017b). This network is also activated upon seed germination, but not during zygotic embryogenesis, indicating that somatic embryogenesis rather resembles the seed germination pathway. Indeed, ectopic expression of the key *BBM*, *LEC1*, or *LEC2* genes results in somatic embryogenesis (Lotan et al., 1998; Stone et al., 2001; Boutilier et al., 2002; Zuo et al., 2002). In seedlings, activity of these transcription factors is epigenetically repressed in order not to interfere with normal development (Holdsworth et al., 2008), as ectopic expression of these transcription factors results in somatic embryogenesis in vegetative tissues, such as cotyledons (Radoeva and Weijers, 2014; Horstman et al., 2017a). Similarly, failure to transcriptionally repress (some of) these key players results in the induction of somatic embryos (Ikeuchi et al., 2015), or using a more artificial tissue-culturing setting, whole plants can be generated from pollen or single protoplasts through somatic embryogenesis (Takebe et al., 1971; Zhu et al., 1997; Maraschin et al., 2005; Chupeau et al., 2013).

Besides the activity of key transcription factors, accumulating evidence indicates that the transcription of many reprogramming genes involved in regeneration and somatic embryogenesis are epigenetically regulated. Specific histone modifications play important roles in determining the activation or repression of gene expression (Ikeuchi et al., 2019). For example, expression of the aforementioned embryonic regulators *LEC2* and *BBM*, and *WIND3*, is developmentally repressed by the evolutionary conserved POLYCOMB REPRESSIVE COMPLEX 2 (PRC2), in order to prevent spontaneous somatic cellular dedifferentiation, callus formation and ectopic onset of embryogenesis, as observed in single root hair cells in *prc2*-deficient plants (Chanvivattana et al., 2004; Bouyer et al., 2011; Ikeuchi et al., 2019). Contrary, HISTONE ACETYL TRANSFERASE OF THE GNAT/MYST SUPERFAMILY (HAG1), also known as GENRAL CONTROL NONREPRESSED 5, plays a pivotal role in the acquisition of shoot regeneration competence. By facilitating histone acetylation and subsequent activation of gene expression, HAG1 is thought to be responsible for activating the expression of *WOX5* and *SCR* in order to confer cellular pluripotency (Kim et al., 2018).

## Putting Things to Use: Tissue Culturing for Genetic Transformation

Genetic modification allows for the generation of new varieties with improved traits *via* knowledge transfer from model to crop species. Central in the search for an efficient transformation system is the need for tissue that is susceptive to transformation and capable of whole plant regeneration. In Arabidopsis, these demands are met by the female gametes, allowing easy and rapid transformation using the Agrobacterium-mediated floral dip method (Clough and Bent, 1998), whereas many other plant species require an elaborate tissue-culturing period as to provide proliferative cells with the capacity to regenerate (Kausch et al., 2019). Important food crops, such as maize, rice or wheat, are monocotyledonous plants and are especially recalcitrant to *in vitro* culturing, conceivably due to their scattered vascular structures that lack the meristematic cell types that are susceptible to culturing (Benson, 2000). Consequently, cereal transformation is a labor- and time-intensive process with a generally low efficiency, which is genotype and explant dependent (Ji et al., 2013). Most reported transformation protocols require the use of immature embryos (IE) or IE-derived callus, for example for the transformation of maize (Ishida et al., 2007; Frame et al., 2011), rice (Hiei and Komari, 2008), and wheat (Ishida et al., 2015). However, the frequency of embryogenic callus induction and further regeneration into transgenic plants is strongly influenced by the tissue-culture media components and culturing conditions.

A more generic, cultivar- and even species-independent approach to improve tissue culturing is to utilize the aforementioned genetic factors from Arabidopsis that play a role in callus formation and following plant regeneration, with a special preference toward factors whose overexpression results in somatic embryogenesis. For example, orthologs of the Arabidopsis embryonic regulators LEC1 and LEC2 have been shown to improve transformation efficiency in maize and wheat (Lowe et al., 2003). Similarly, orthologs of SOMATIC EMBRYOGENESIS RECEPTOR KINASE (SERK) originally identified in carrots (Schmidt et al., 1997) have been linked with somatic embryogenesis in maize, rice, and rye (Baudino et al., 2001; Singla et al., 2009; Gruszczyńska and Rakoczy-Trojanowska, 2011). Recently, also the maize ortholog of the developmental regulator GROWTH-REGULATING FACTOR 5 (GRF5), and the wheat ortholog of GRF4 together with its cofactor GRF-INTERACTING FACTOR 1 (GIF1) have been linked with increased regeneration (Debernardi et al., 2020; Kong et al., 2020). Many transformation protocols currently available rely on the embryonic regulators BBM and WUS. Combined *BBM* and *WUS* overexpression leads to growth stimulation of embryogenic tissue in recalcitrant maize, rice, and sorghum, and expands the range of successful explants from IE or IE-derived callus to mature seeds and even leaf tissue (Lowe et al., 2016). However, there is a trade-off as constitutive expression of these factors results in developmental defects and therefore needs to be excised from engineered plants. Therefore, the recently reported GRF-GIF module may emerge as an interesting alternative. GRFs and GIFs interact to form a complex, therefore combined expression can be used to drastically improve regeneration efficiencies, as observed in *GRF4-GIF1* expressing “chimeras” from wheat (Debernardi et al., 2020). The GFR-GIF chimera approach appears to work efficiently to increase regeneration in both monocotyledonous species (including durum wheat, common wheat, rice, and triticale, being a hybrid between wheat and rye), as well as dicotyledonous species, including citrus and, contrary to BBM-WUS, does not result in developmental defects (Luo and Palmgren, 2021).

## Conclusion

Researchers have been drawn to explore and utilize the regenerative power of plants for more than 100 years (Haberlandt, 1902). Although a vast number of phenotypic responses and molecular data have already been gathered to date, plants have not revealed all their regenerative secrets yet. The further fine-tuning of microscopic techniques, such as laser-mediated cell ablation, and the development of high-resolution single cell transcript profiling have provided the possibility to investigate new and more detailed aspects of the plants’ response following tissue damage. Ranging from a plant that needs to regenerate a single cell to a single cell that needs to regenerate a plant, and everything in between, nothing appears to be impossible. This once again highlights that even a differentiated plant cell possesses the capacity to regain the potential to form all different cell types that constitute a plant, with or without a minor biotechnological intervention. However, some crop species or genotypes remain more recalcitrant when it comes down to regeneration efficiency or tissue culturing. For many crops, tissue culturing is still an inherent step to genetic modification. Central in the search for an efficient genome editing system is the need for an accessible and susceptible tissue that is receptive to transformation and capable of subsequent regeneration into fertile plants. Finding the key to unlock *in vitro* culturing, regardless of the explant tissue used, or the full regeneration potential of recalcitrant (crop) species, will undoubtedly have a significant impact on their transformation efficiency and agri- and horticultural applications.

## Author Contributions

JH, FC, BC, FL, and LDV wrote the manuscript. AB and SS provided critical suggestions for the manuscript. JH created the figures. All authors contributed to the article and approved the submitted version.

## Conflict of Interest

The authors declare that the research was conducted in the absence of any commercial or financial relationships that could be construed as a potential conflict of interest.
